# A proposed amendment to the current guidelines for mild traumatic brain injury: reducing computerized tomographies while maintaining safety

**DOI:** 10.1007/s00068-019-01145-x

**Published:** 2019-05-14

**Authors:** Tomas Vedin, Mathias Karlsson, Marcus Edelhamre, Linus Clausen, Sebastian Svensson, Mikael Bergenheim, Per-Anders Larsson

**Affiliations:** 1 Clinical Sciences, Helsingborg, Lund University, Svartbrödragränden 3-5, 251 87 Helsingborg, Sweden; 2 Department of Clinical Chemistry, Center for Clinical Research, Centralsjukhuset, Karlstad, Sweden; 3 Centralsjukhuset i Karlstad, Rosenborgsgatan 9, 652 30 Karlstad, Sweden

**Keywords:** Brain injuries, Traumatic, Tomography, X-ray computed, Practice guidelines as topic, Intracranial hemorrhage, Traumatic

## Abstract

**Purpose:**

Head trauma is a common complaint in emergency departments. Identifying patients with serious injuries can be difficult and generates many computerized tomographies. Reducing the number of computerized tomographies decreases both cost and radiation exposure. The aim of this study was to evaluate whether the current Scandinavian Neurotrauma Committee guidelines could be revised in such a way that would enable hospitals to perform fewer computerized tomographies while maintaining the ability to identify all patients requiring neurological intervention.

**Methods:**

A retrospective study of the medical records of adult patients suffering a traumatic brain injury was performed. A total of 1671 patients over a period of 365 days were included, and 25 parameters were extracted. Multitrauma patients managed with ATLS™ were excluded. The Scandinavian Neurotrauma Committee guidelines were amended with the previously derived “low-risk proposal” and applied retrospectively to the cohort.

**Results:**

Incidence of intracranial hemorrhage was 5.6% (93/1671). Application of the current Scandinavian Neurotrauma Committee guidelines would have resulted in 860 computerized tomographies and would have missed 11 intracranial hemorrhages. The proposed amendment with the low-risk proposal would have resulted in 748 CT scans and would have missed 19 intracranial hemorrhages (a relative reduction of 13%). None of the missed intracranial hemorrhages required neurological intervention.

**Conclusion:**

For patients with mild and moderate traumatic brain injuries, application of the Scandinavian Neurotrauma Committee guidelines amended with the low-risk proposal may result in a significant reduction of computerized tomographies without missing any patients in need of neurological intervention.

## Introduction

Traumatic brain injury continues to be a topic of interest for researchers worldwide, because it is a major cause of morbidity, mortality, and emergency department visits. Furthermore, it presents challenges for physicians in the emergency setting, because patients’ initial signs and symptoms do not always correlate with the extent of brain injury.

The epidemiology of traumatic brain injury has changed over the past decade with both increasing age of patients and different mechanisms of trauma. The adult patients that sustain traumatic brain injury appear to be getting older. We previously reported a median age of 58 years, and this was congruent with studies published over the last 5 years [1–3]. In earlier studies, the incidence was lowest at around 55 years. Moreover, the most common trauma mechanism has changed from motor vehicle accidents to falls [4–10]. The shift in epidemiology has coincided with a shift in medications affecting coagulation and thrombocyte function. Our previous study pointed to an increased incidence of intracranial hemorrhage in patients taking platelet inhibitors compared to patients on anticoagulants. The intracranial hemorrhage incidence was 8.6% (10/116) in anticoagulated patients and 11.8% (20/169) among those on platelet inhibitors [1].

**Fig. 1:**
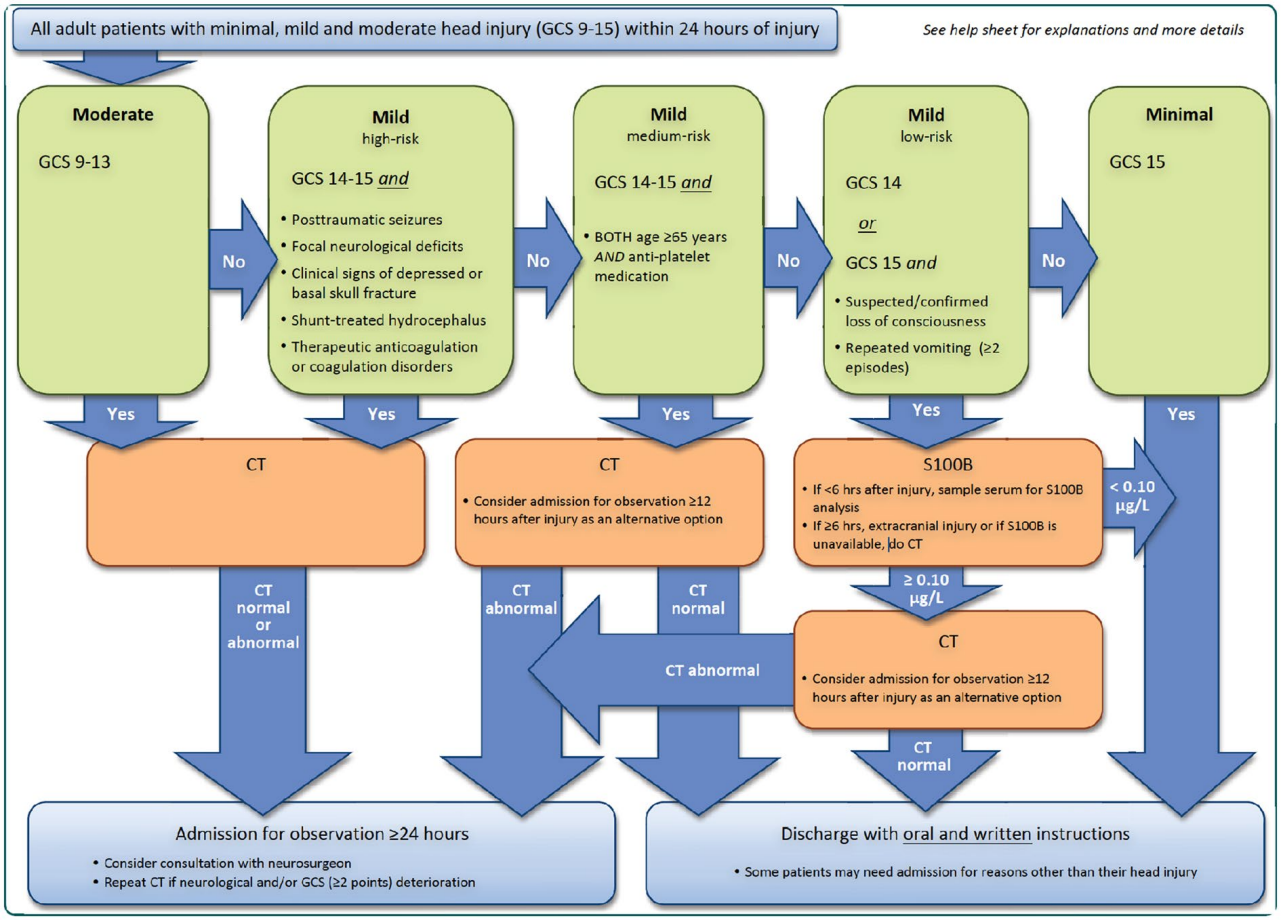
Title: Scandinavian Neurotrauma Committee Guideline for adult patients with minimal mild and moderate head injury. Reprinted with the permission of author Johan Undén, Scandinavian Neurotrauma Committee

Several sets of guidelines for the emergency management of traumatic brain injury are available. Among some of the most widespread are the Canadian CT Head Rule (CCHR), the New Orleans Criteria (NOC), and the National Institute for Health and Care Excellence (NICE) guidelines [11–13]. Furthermore, the Scandinavian Neurotrauma Committee (SNC) guidelines were the first to incorporate brain biomarker S100B. See Fig. 1 for outline of the SNC Guideline [14]. S100B originates from astrocytes and controls calcium homeostasis [9]. In vitro, it has neurotrophic effects in nanomolar concentrations and neurotoxic effects in micromolar concentrations [15]. It is secreted into cerebrospinal fluid, passes into the blood, and is eliminated in the kidney. It has a half-life of 25–97 min [16, 17]. Current guidelines are based on prospective data gathered in the 1990s or on meta-analyses [11–14]. Because of the changes in trauma mechanism, age, anticoagulation, and thrombocyte inhibitors, it is conceivable that guidelines based on newer data could be even more accurate.

Previous studies have reported an intracranial hemorrhage incidence of 4–8% and a neurosurgical intervention incidence of below 10% in patients with intracranial hemorrhage [11, 18–20]. When correctly applied, both the CCHR and NOC lead to slightly more than 50% of patients being scanned with computerized tomography (CT) of the head [21]. The CCHR guidelines are constructed chiefly to rule out intracranial hemorrhage requiring intervention and have been validated with a 100% detection rate [21–23]. The SNC guidelines aim to detect all intracranial hemorrhages and have been prospectively validated as safe [18]. In the validation study of the SNC guidelines, CT was recommended in 68.1% of cases. Repeated head CTs with increased cumulative radiation dose may have cancerogenic effects and should, therefore, be avoided when possible [24–27]. It can also be argued that identifying intracranial injuries requiring intervention is the most important purpose of a guideline. Taking into account possible cancerogenic effects, emergency room overcrowding, and the health economy, a safe guideline that recommends CTs in less than 50% of cases would benefit patients, emergency department staff, and the economy.

We previously presented data that indicated the presence of a large group of patients with head trauma that could possibly have been safely discharged based on trauma energy level obtained from patient history, regardless of signs and symptoms. This subgroup included patients 58 years or younger, without anticoagulants or platelet inhibitors subjected to low-energy trauma [1]. This finding will be referred to as the “low-risk proposal”.

The primary aim of the current study was to investigate an amendment of the Scandinavian Neurotrauma Committee guidelines based on the low-risk proposal that might enable hospitals to perform fewer computerized tomographies while maintaining the ability to identify all patients requiring neurosurgical intervention. The secondary aim was to evaluate intracranial hemorrhage incidence in patients taking thrombocyte inhibitors compared those taking oral anticoagulants.

## Materials and methods

The study was performed as a retrospective analysis of medical records in patients with isolated head trauma in the emergency department at Helsingborg General Hospital. The hospital catchment area of 350,000 people generates about 75,000 emergency department patient visits each year. Neurosurgical care is provided at Skane’s University Hospital in Lund, 40 km away. Multitrauma patients were managed according to ATLS™. The in-hospital recommended guidelines for traumatic brain injury during the study period were the SNC guidelines.

The inclusion criteria were classification as “head trauma” by an emergency department triage nurse and an age of 18 years or older. The exclusion criteria were multitrauma, check-up visits, and management by triage nurse only. After extracting a list of all patients satisfying the inclusion criteria, a medical record review was performed. Patients may have had additional minor injuries, but if they were initially classified as multitrauma (*n* = 917), they were excluded. This exclusion was performed to make the cohort representative of emergency department patients with minor traumatic brain injury. Multitrauma patients frequently require head CT scans based on ATLS™ criteria, and these patients fell outside the scope of evaluation. The review was performed on patients registered between January 1, 2017, and December 31, 2017, a total of 365 days.

Hospitals in Sweden use the Reaction Level Scale (RLS) instead of the Glasgow Coma Scale [28]. It is difficult to assess the Glasgow Coma Scale level retrospectively. Thus, degree of consciousness was reported on the basis of the RLS throughout the present study.

The following parameters pertaining to this article were manually extracted from medical records:

1. Age (years).
2. Gender (male/female).
3. Head CT performed (yes/no).
4. Head CT outcome (hemorrhage/no hemorrhage).
5. Admission to general hospital ward (yes/no).
6. Admission to intensive care unit or neurointensive care unit (yes/no).
7. Neurological intervention (yes/no).
8. Degree of head injury (minimal, mild, moderate, severe).
9. Level of consciousness using Reaction Level Scale 85 (1–8).
10. Past illnesses (yes/no).
11. Anticoagulant treatment (no/warfarin/novel anticoagulant/low molecular weight heparin).
12. Platelet inhibitor treatment (no/aspirin/clopidogrel/ticagrelor/prasugrel/dipyramidol/combinations).
13. Other medication (yes/no).
14. New focal neurological deficits (yes/no).
15. S100B level (μg/l).
16. Nausea (yes/no).
17. Vomiting (yes/no).
18. Number of vomits (number).
19. Amnesia, type, and duration (yes/no, antegrade/retrograde, time hh:mm).
20. Loss of consciousness (yes/no).
21. Peritraumatic seizure (yes/no).
22. Trauma energy level (low, medium, high).
23. Trauma mechanism.
24. Clinical signs of basal skull fracture and/or skull fracture (yes/no).
25. Influence of any or multiple drugs/alcohol (yes/no, alcohol, sedatives, and central stimulants).

Level of trauma energy (see #22 above) was interpreted on the basis of trauma mechanism, as follows:

- Low [11]: fall from less than 1 m or fewer than five stairs.
- Medium [11]: fall from 1 to 3 m or five or more stairs.
- High (ATLS™): fall from 3 m or more, motor vehicle accident at 70 km/h or more with seatbelt, motor vehicle accident at 30 km/h or more without seatbelt, any motorcycle accident, pedestrian hit by motor vehicle.

Minimal traumatic brain injury was defined as trauma to the head without loss of consciousness and without any of the following criteria: amnesia, nausea, vomiting, vertigo, or focal neurological deficits [29].

Mild traumatic brain injury was defined as acute brain injury due to mechanical energy to the head from external physical forces. Criteria for clinical identification were as follows:

1. One or more of the following: confusion or disorientation, loss of consciousness for 30 min or less, posttraumatic amnesia for less than 24 h, and other transient neurological abnormalities such as focal signs, seizure, and intracranial lesion not requiring surgery.
  and
2. RLS score of 1–2 either 30 min after injury or when subsequently examined by a health-care professional [6].

Moderate traumatic brain injury was defined as a brain injury resulting in loss of consciousness from 20 min to 6 h and an RLS score of 3 [30].

Severe traumatic brain injury was defined as a brain injury resulting in loss of consciousness for more than 6 h and an RLS score of 4–8 [30].

Neurological intervention was defined as either death within 7 days due to brain injury or any of the following procedures within 7 days: intubation for head injury shown on CT, intracranial pressure monitoring, elevation of skull fracture, or craniotomy [11].

A known problem with retrospective review of medical records is information bias. To minimize this bias, we followed the guidelines for retrospective review developed by Vassar and Holzmann [31]. To further increase validity, all medical records were reviewed by one researcher and a randomized sample of 100 medical records was reviewed by another researcher to calculate the interrater reliability score (Cohen’s kappa coefficient). Cohen’s kappa was calculated for parameters commonly associated with increased risk of intracranial hemorrhage. These parameters included trauma mechanism, previous diseases, intoxication, loss of consciousness, amnesia, number of vomits, posttraumatic seizures, new neurological deficits, thrombocyte inhibitor treatment, and anticoagulation treatment.

### Statistics

Data was analyzed using SPSS version 25 for Mac. Statistical significance was set at *p* < 0.05. Histograms were used to test for normal distribution. Central tendencies were presented as medians when skewed. Descriptive statistics were applied to delineate the cohort. Guideline performance was tested with sensitivity, specificity, negative predictive value, and positive predictive value. Contingency tables were tested using the *χ*^2^ test (*n* > 5).

Cohen’s kappa coefficient was calculated to assess agreement between the two medical record reviewers. To make a good estimate of the agreement without having the second reviewer examine all the records, a sample was used. The sample size was calculated assuming that the raters would agree in 50% of cases and that agreement between reviewers would differ no more than 20% from the value of the whole population (relative error). Satisfying these conditions, the sample size was calculated at 93 medical records and the second reviewer examined 100 medical records.

### Ethics

The study was approved by the Regional Ethical Review Board in Lund. It was conducted in accordance with the Helsinki Declaration. Specific national laws were observed.

## Results

A total of 1671 patients met the inclusion criteria and were included in the analyses. The median age was 64 years (18–104, IQR 41 years), 887/1671 (53.1%) of the participants were male, and 784 (46.9%) were female.

Intoxication was found in 385/1671 (23%) patients; 359/385 (93.2%) were intoxicated with alcohol and 26/385 (6.8%) with other or multiple substances.

Of these 1671 patients, 756 (46.1%) had minimal head injury, 894 (53.5%) had mild head injury, 11 (0.7%) had moderate head injury, and 10 (0.6%) had severe head injury.

Level of consciousness was RLS 1 in 1588/1671 (95.0%) cases, RLS 2 in 62/1671 (3.7%) cases, and RLS 3 in 11/1671 (0.7%) cases, and 10/1671 (0.6%) patients were unconscious with varying degrees of level of consciousness (RLS 4–8).

Trauma energy level was low in 1033/1671 (61.8%) patients, whereas 38/1671 (2.3%) patients had medium trauma energy and 9/1671 (0.5%) had high trauma energy. Of the remaining patients, 500/1671 (29.9%) had classifiable trauma mechanisms without trauma energy classification, and 89/1671 (5.3%) had an unclear trauma mechanism.

S100B was measured in 434/1671 (26%) cases in the entire cohort. The median S100B level was 0.12 μg/l (IQR 0.15 μg/l, range 0.02–2.60 μg/l). S100B was measured in 88/242 (36.4%) cases in the low-risk-proposal cohort, in which the median S100B level was 0.09 μg/l (IQR 0.11 μg/l, range 0.03–0.68 μg/l).

During the study period, 1039/1671 (62.2%) patients underwent CT of the head. The SNC guidelines recommended CT in 860 patients. Additionally, 179 patients underwent CT without guideline recommendation. The median age of patients who underwent CT was 73 years (18–101 years, IQR 32 years), and that of patients who did not undergo CT was 44 years (18–100 years, IQR 38 years). All patients with moderate and severe head injury underwent CT.

Total CT-verified intracranial hemorrhage incidence was 5.6% (93/1671 patients). The distribution of intracranial hemorrhage was 54/93 (58%) in males and 39/93 (42%) in females. The distribution of intracranial hemorrhages in different degrees of head injury can be found in Table 1.

**Table 1:** Intracranial hemorrhage by severity of head injury

Head injury severity	Intracranial hemhorrage		
	No	Yes	%
Mild	1569	81	4.9
Moderate	6	5	45.5
Severe	3	7	70.0
Total number	1578	93	5.6

The rate of admission to the surgical ward was 21.5% (360/1671 patients), and 11/1671 (0.7%) patients were admitted to the intensive care unit. Neurological intervention was performed in 8/1671 (0.5%) patients. Mortality was 0.5% (8/1671).

The low-risk-proposal cohort consisted of 242/1671 (14.5%) patients, of which eight patients (six females and two males) had intracranial hemorrhage (ages 21, 43, 44, 48, 48, 51, 54, and 55 years). All but two females (aged 51 and 54 years) were intoxicated, and all but three females (ages 48, 54, and 55 years) had past illness. Only the 54-year-old female was sober and had no past illness. None of the patients in this cohort required neurological intervention.

The SNC guidelines recommended 860/1671 (51.5%) CTs, correctly diagnosed 82/93 intracranial hemorrhages (89.2%), and missed 11 intracranial hemorrhages in the 179 patients who were CT scanned against guideline recommendations. The SNC guidelines amended on the basis of the low-risk proposal recommended 748/1671 (44.8%) CTs, correctly diagnosed 74/93, and missed 19 intracranial hemorrhages diagnosed with CT performed according to or against the SNC guidelines. Neither the original nor the amended version of the guidelines missed intracranial hemorrhages that required neurological intervention (Tables 2, 3).

**Table 2:** Scandinavian Neurotrauma Committee guidelines applied to entire cohort

Degree of head injury	Total number (*N*)	Computerized tomographies (*N*)	Intracranial hemorrhages (*N*)	Interventions (*N*)	Missed intracranial hemorrhages (*N*)	Missed intracranial hemorrhages with intervention (*N*)
Severe injury	10	10	7	2	0	0
Moderate injury	11	11	5	2	0	0
Mild injury, high risk	433	433	29	3	0	0
Mild injury, medium risk	142	142	14	1	0	0
Mild injury, low risk (RLS2)	41	36	2	0	0	0
Mild injury, low risk (RLS1)	278	228	25	0	0	0
Minimal injury	756	0	11	0	11	0
Total number (*N*)	1671	860	93	8	11	0

**Table 3:** Amended Scandinavian Neurotrauma Committee guidelines applied to entire cohort

Degree of head injury	Total number (*N*)	Computerized tomographies (*N*)	Intracranial hemorrhages (*N*)	Interventions (*N*)	Missed intracranial hemorrhages (*N*)	Missed intracranial hemorrhages with intervention (*N*)
Severe injury	10	10	7	2	0	0
Moderate injury	11	11	5	2	0	0
Mild injury, high risk	433	392	29	3	3	0
Mild injury, medium risk	142	142	14	1	0	0
Mild injury, low risk (RLS2)	41	29	2	0	0	0
Mild injury, low risk (RLS1)	278	164	25	0	5	0
Minimal injury	756	0	11	0	11	0
Total number (*N*)	1671	748	93	8	19	0

Application of the low-risk proposal resulted in a relative reduction of 13% of head CTs (112 CTs). The reduction of CTs was statistically significant (*p* < 0.001). The specificity was raised from 50.7 to 57.3%, and the negative predictive value was lowered from 98.6 to 97.9% (Table 4).

**Table 4:** Performance of Scandinavian Neurotrauma Committee guidelines and amended Scandinavian Neurotrauma Committee guidelines

Type of performance measurement	Scandinavian Neurotrauma Committee guidelines	Amended Scandinavian Neurotrauma Committee guidelines
Sensitivity	88.2% (95% CI 79.8–94.0%)	79.6% (95% CI 70.0–87.2%)
Specificity	50.7% (95% CI 48.2–53.2%)	57.3% (95% CI 54.8–59.8%)
Negative predictive value	98.6% (95% CI 97.7–99.2%)	97.9% (95% CI 97.0–98.6%)
Positive predictive value	9.5% (95% CI 8.8–10.3%)	9.9% (95% CI 8.9–11.0%)

Of the entire cohort, 194/1671 (11.6%) were being treated with some kind of thrombocyte inhibitor. Aspirin (75 mg) was being taken by 166/194 (86%). Anticoagulant treatment was being given to 215/1671 (12.9%). Warfarin was being taken by 111/215 (51.6%) and novel oral anticoagulants (NOACs) by 104/215 (48.4%). Intracranial hemorrhage incidence was 11.9% (23/194) in the thrombocyte inhibitor cohort and 6.0% (13/215) in the anticoagulant cohort. In the remainder of the cohort, which was without thrombocyte inhibitors, anticoagulants, or low molecular weight heparins (LMWHs), there were 58/1254 (4.6%) intracranial hemorrhages. This distribution was statistically significant (*p* = 0.037). Further subcategorization was made between different drugs in each drug class, but no statistically significant differences were found between aspirin and other thrombocyte inhibitors, between warfarin and NOACs, or among different types of LMWHs.

Cohen’s kappa coefficient varied between 0.167 and 0.857, which in all parameters but “new neurological deficits” and “LMWH treatment” corresponds to good and very good agreement [32]. See Table 5.

**Table 5:** Cohen’s kappa coefficient for signs and symptoms associated with intracranial hemorrhage

Parameter	Cohen’s kappa	95% CI lower	95% CI upper
Trauma mechanism	0.641	0.521	0.761
Previous disease	0.717	0.580	0.854
Intoxication	0.857	0.739	0.975
Loss of consciousness	0.763	0.594	0.932
Amnesia	0.686	0.512	0.860
Two or more vomits after trauma	0.756	0.648	0.864
Posttraumatic seizure	^a^		
New neurological deficits	0.347	0.186	0.508
Thrombocyte inhibitor	0.825	0.702	0.948
Anticoagulant	0.799	0.697	0.901
LMWH treatment	0.167	− 0.168	0.502

^a^Variable is a constant; therefore, it is not possible to calculate kappa coefficient

## Discussion

This study showed that it was theoretically possible to amend the SNC guidelines with the low-risk proposal and achieve significant reduction of head CTs while maintaining the ability to detect all intracranial hemorrhages requiring intervention. The potential benefits of this include reduced cost, reduced radiation dose, and reduced emergency department overcrowding. The cost of a head CT has been estimated at $600–$1200, and the risk of cancer after one head CT in adults to 1/6000–1/12,000 [24, 33]. Emergency room overcrowding is a growing global issue, and the use of standardized routines to manage need of radiography has been suggested as part of the solution [34].

The amended guideline failed to diagnose 4.3–7.3% of intracranial hemorrhages and most clinicians might at first be reluctant to miss as many as 7.3%. However, we suggest that the absolute number of missed hemorrhages is of less importance. It is rather the characteristics and subsequent consequences of the hemorrhages that are relevant. High-level evidence on what intracranial hemorrhage characteristics that predict neurological intervention is lacking [20, 35–38]. Because of this, we maintain that the most pragmatic way to quantify the undiagnosed intracranial hemorrhages of the amended guideline is by “intervention/no intervention” and not by characteristics such as type, size or rate.

Better evidence would enable development of head trauma guidelines that aim at only recommending CTs for patients with an actual risk for intervention. If all hemorrhages requiring intervention were found by such a guideline, there would be no safety issue and the number of CTs could possibly be reduced. Nonetheless, this might lead to another clinical problem. Intracranial hemorrhage is associated with increased incidence of postconcussion syndrome and postconcussion headache [39, 40]. Treatment is available for these patients, but if they are not identified by emergency head CT, another means of identifying them is necessary so they can be offered treatment [41, 42]. A tentative approach would be to provide patients with detailed written information and provide them with an easy way back into the medical system. Many patients with persisting symptoms return to the emergency room or seek primary care, and both the patient and the medical system could benefit from having this second workup performed during office hours. If radiology was needed as part of the second workup, magnetic resonance imaging could be performed instead of CT, consequently eliminating radiation, placing less stress on the emergency radiology department, and providing more accurate diagnosis of the pathologies that do not require neurosurgical intervention but still some other kind intervention such as physical therapy [43]. Another approach to finding those in need of neurorehabilitation could be other biomarkers than S100B. A recent study demonstrated that glial fibrillary acidic protein and ubiquitin c-terminal hydrolase L1 can be used in combination to safely both rule in and rule out intracranial hemorrhage and to some extent predict the need for neurological intervention [44]. Because of the high sensitivity of these biomarkers for intracranial hemorrhage, maybe they can be used to identify the patients that will develop postconcussion syndrome as well.

The low-risk-proposal cohort in the present study was significantly smaller than that in our previous study (242/1671 patients versus 826/1641 patients; *p* < 0.05) [1]. We believe this discrepancy can be explained at least in part by our more detailed review of the trauma mechanisms. It is possible that more patients were classified as low-energy trauma in the previous study because the trauma mechanism variable had fewer alternatives. The current study’s more comprehensive review of trauma mechanisms improves the validity of results pertaining to our primary aim of evaluating the low-risk proposal.

The 179 CT scans performed against the SNC recommendations resulted in the identification of an additional 11 intracranial hemorrhages not requiring neurological intervention. The value of diagnosing these intracranial hemorrhages is unclear, because it might lead to unnecessary hospitalization and repeated CT scans. None of these CTs showed a pathology that required neurological intervention. In spite of validated, hospital-recommended guidelines, physicians continue to prescribe unwarranted CT scans. This is in accordance with our previous study which was performed at the same emergency department. It demonstrated that physicians trust their own judgment over guidelines, but order CT scans even when they rate the probability of intracranial hemorrhage as low. The guideline adherence in this study was between 40 and 60%, which was in line with similar studies. It is a known problem that guideline adherence sometimes might be too low for improvement of guidelines to have any real impact. This problem needs to be addressed simultaneously as introduction of an amended guideline to ensure that the guideline will be as useful as possible [45].

In the present study, the doubled rate of intracranial hemorrhage among patients taking thrombocyte inhibitors compared with patients taking anticoagulants is consistent with the trend shown in our previous study as well as that shown by Nishijima et al. [1, 46]. In our previous study, the hemorrhage rate in the thrombocyte inhibitor group was 50% higher and only a few of the anticoagulated patients had NOACs. The reduced incidence of intracranial hemorrhage in anticoagulation cannot be explained in this study by an increased number of patients taking NOACs, because there were no statistically significant differences between these groups. However, these findings are in agreement with studies on the safety of NOACs versus warfarin [47, 48], and it is feasible that the cohort was too small to detect a possible difference. This reinforces our conviction that future guidelines should treat thrombocyte inhibitors and anticoagulants as equally dangerous and should recommend CT for all head trauma patients taking either of these drugs.

The performance of S100B is similar to that shown by previous studies and indicates that even though it correctly classifies most patients with intracranial hemorrhage, it is not perfect [49]. Therefore, it must be used with some caution, and the value of good clinical judgement should be taken into account when designing new guidelines.

A retrospective study has limitations. We tried to limit information bias by observing specific guidelines for retrospective research and by conducting interrater reliability tests that showed overall high concordance between raters [31]. Based on the present study, we cannot recommend management based on the low-risk proposal. However, we recommend prospective testing of this amended guideline and propose that both thrombocyte inhibitor treatment and anticoagulant treatment should be treated as exposing patients to a high risk of intracranial hemorrhage.

## Conclusion

Application of the SNC guidelines amended on the basis of the low-risk proposal among patients with mild and moderate traumatic brain injuries may result in a significant reduction of CTs without missing any patients who require neurological intervention. We cannot recommend clinical management based on the low-risk proposal, but these findings merit prospective testing. We also propose that thrombocyte inhibitor treatment and anticoagulant treatment should be regarded as carrying an equally high risk of intracranial hemorrhage.
